# G protein-coupled receptor kinase 4 governs early kidney development via STAT3 and Nhe3a

**DOI:** 10.3892/ijmm.2026.5953

**Published:** 2026-08-11

**Authors:** Linh Anna Trúc Vu, Julian Gerhards, Lars D. Maerz, Martin D. Burkhalter, Melanie Philipp

**Affiliations:** 1 Department of Experimental and Clinical Pharmacology and Pharmacogenomics, Section of Pharmacogenomics, Eberhard-Karls-University Tübingen, D-72074 Tübingen, Germany; 2 Institute of Biochemistry and Molecular Biology, Ulm University, D-89081 Ulm, Germany; 3 Department of Forensic Toxicology, Institute of Legal Medicine, University Medical Center Mainz, D-55131 Mainz, Germany

**Keywords:** G protein-coupled receptor kinase 4, zebrafish, kidney development, Nhe3, cilia, dopaminergic receptor

## Abstract

G protein-coupled receptor kinase 4 (GRK4) is a member of the receptor phosphorylating GRK family with a well characterized function in renal sodium reabsorption through the modulation of dopaminergic receptors in adults. During embryonic development, GRK4 controls renal cilium development and thus, kidney function. The loss of GRK4 in zebrafish embryos results in pronephric dilatation, massive cilium elongation, glomerular cyst formation and, as a consequence of renal dysfunction, brain edema such as hydrocephalus. Notably, these embryonic functions of GRK4 appear to be independent of G protein-coupled receptor modulation, although the full extent of the abilities of GRK4 to modulate cellular signaling has remained unclear. The present study aimed to provide further insight on this aspect of the functions of GRK4. Using zebrafish embryos, the present study demonstrates that GRK4 is required for normal pronephros patterning. The loss of Grk4 resulted in the upregulation of the sodium-hydrogen exchanger 3a (Nhe3a), the zebrafish homologue of human NHE3. The administration of the NHE3 inhibitor, tenapanor, restored normal kidney development and function. Of note, during embryogenesis, this modulation of Nhe3a is not connected to dopaminergic receptor signaling or to a possible nuclear function of GRK4. Instead, the present study identified elevated Stat3 activity as the underlying cause leading to increased Nhe3a transcription and subsequently, to defects in kidney development.

## Introduction

G protein-coupled receptor kinase 4 (GRK4) is one of seven kinases originally identified for their regulatory activity at ligand-stimulated G protein-coupled receptors (GPCRs) (1). GRK4, which belongs to the GRK4-6 subfamily, is the least investigated GRK, as it was initially only detected in a small number of tissues and due to the fact that whole-body knockout mice are viable (2,3). With the aid of more sensitive methods than those originally used, it is currently known that GRK4 is almost ubiquitously expressed (4). Moreover, the other GRKs of its subfamily appear to compensate for the loss of GRK4, rendering knockout mice apparently indistinguishable from wild-type mice (2,4). To date, only a limited amount of research has been performed on the abilities of GRK4 to modulate cellular signaling, apart from certain functions during renal sodium reabsorption. The identification of genetic variants associated with salt-sensitive hypertension sparked research on GRK4 and revealed a substantial involvement of GRK4 in the regulation of renal dopaminergic receptors, besides others. Recently, the authors expanded on this knowledge and developed loss-of-function (LOF) models of Grk4 in zebrafish, which revealed that Grk4 has functions in the developing kidney. The loss of Grk4 in zebrafish embryos results in uncontrolled cilium elongation, pronephric tubule dilatation and glomerular cyst formation. Together, these disrupt the functionality of the developing kidney, as evidenced by proteinuria and edema formation, such as hydrocephalus (4). These phenotypes further mimic human ciliopathies characterized by polycystic kidneys (5).

Notably, the early embryonic functions of Grk4 appear to be largely independent of dopamine receptor regulation. Instead, it has been demonstrated that GRK4 limits mTOR complex 1 activation, which has similarly been shown for GRK5 (6,7). The pharmacological inhibition of mTOR activity, however, only partially rescued the phenotypic spectrum suggesting that Grk4 impacts on cellular signaling via additional mechanisms (4).

The present study expanded on the previous study by the authors (4) and demonstrates that the loss of Grk4 function disrupts normal pronephric patterning and increases expression of the sodium-hydrogen exchanger 3a (Nhe3a) independently of dopaminergic receptors. The decrease in the expression of Nhe3a ameliorates the phenotypes induced by Grk4 loss-of-function (LOF). Grk4 induces *nhe3a* expression in a receptor-independent manner through the activation of signal transducer and activator of transcription 3 (STAT3), a protein known to augment NHE3 expression in renal epithelial cells (8). Interference with increased Stat3 signaling in zebrafish normalizes *nhe3a* expression, hydrocephalus and cyst formation, suggesting that a Stat3-Nhe3a axis contributes to the development of phenotypes induced by Grk4 LOF.

## Materials and methods

### Reagents

Dopamine HCl (cat. no. H8502), fenoldopam (cat. no. SML0198), phenylthiourea (cat. no. P7629) and proteinase K (cat. no. 3115836001) were obtained from Merck KGaA, SCH39166 hydrobromide was purchased from Biomol (cat. no. Cay25331), and tenapanor was purchased from BIOZOL Diagnostica Vertrieb GmbH (cat. no. ADQ-A14011-2). Poly-D-lysine was obtained from Merck KGaA. Veterinarian-grade tricaine mesylate was obtained from PharmaQ.

### Zebrafish handling and manipulation

The following fish lines were used: AB and EK wild-type lines, Grk4^mut^ (4) and *Tg(wt1b:GFP)* (9). Adult zebrafish were housed in a Zebtec zebrafish holding rack system with removable tanks, permanent water cleanup and recycling and automatic monitoring and adjustment of water parameters such as pH, temperature and conductivity (Tecniplast). The fish were kept under a 14-h light and 10-h dark cycle and were fed three times a day with pelleted dry food (Sparos) and once a day with living artemia. Zebrafish husbandry and propagation were approved by local authorities at Ulm University and University of Tuebingen, as well as the regional board for scientific animal experiments. Experiments performed herein did not require an additional permit and were carried out in agreement with the European Communities Council Directive of September 22, 2010 (2010/63/UE).

Fertilized eggs were generated by natural mating and embryos were incubated at 28.5°C in embryo medium (13.7 mM NaCl, 0.5 mM KCl, 0.025 mM HNa_2_PO_4_, 0.044 mM KH_2_PO_4_, 1.3 mM CaCl_2_, 1 mM MgSO_4_ × 7 H_2_O, 0.42 mM NaHCO_3_, pH 7.2) until the desired stage. Manipulation by microinjection was performed at the 1-cell stage using an antisense morpholino oligonucleotide (MO) targeting the translation start site of zebrafish Grk4 as previously characterized and published (4): Grk4 MO: 5'-GTC AGA AGA AAG ATC CCA TGC AGT C. All injected embryos were controlled against embryos injected with a control MO (CTRL MO: 5'-GTG ACA ACA AAC ATC CCA TCC AGT C) harboring 5 mismatches compared to the Grk4 MO (4). In addition, uninjected wild-type embryos of the same clutch were incubated along to assess clutch quality. All MOs were custom-synthetized by Gene Tools.

Zebrafish embryos homozygous for a hypomorphic allele of Grk4 (named Grk4^mut^) were generated by heterozygous mating. Embryos were raised until the desired stage, which was either 20 somite stage or 48 hpf, processed for analysis and subsequently genotyped (please see below).

Manipulations with small chemical compounds were performed from the tailbud stage until the final analysis was completed. All compound treatments were controlled against the vehicle DMSO, in which the compounds were dissolved. Fenoldopam, SCH39166 and tenapanor were dissolved in DMSO and used at 5 *μ*M (tenapanor) and 10 *μ*M (fenoldopam, SCH39166) final concentration.

Embryos undergoing subsequent fluorescent analyses were furthermore treated from tailbud stage with phenylthiourea to block pigmentation. Analyses (i.e. image analysis or phenotypic scoring) did not include the blinding of the experimenter.

### Genotyping of Grk4^mut^ embryos

Embryos were digested in lysis buffer (100 mM Tris HCl pH 8.5, 5 mM EDTA, 0.2% SDS, 200 mM NaCl) containing 1 mg/ml proteinase K at 55°C overnight. An equal amount of isopropanol was added and the DNA was pelleted by centrifugation at room temperature, 11,000 × g for 15 min. The DNA pellet was dissolved in ultrapure water and directly used for polymerase chain reaction (PCR). PCR was performed with the following primers amplifying exon 7 along with parts of the flanking introns selected based on the Ensembl sequence ENSDART00000066638.7: WT forward, 5'-TGGGATTTACGGTGCATTGTTATTAGTGAA and Mut forward, 5'-GACATTACAGGGTATTGGGCGGAT and reverse, 5'-ACCACTGATAACGCCCATTCAATCAAATG, and Taq polymerase from NEB (cat. no. M0267). Following an initial denaturation at 94°C for 1 min, 35 cycles of 12 sec 94°C, 15 sec 68°C and 30 sec elongation at 68°C were run followed by an additional 3 min at 68°C. Bands (WT: 493 bp, mutant: 487 and 385 bp) were resolved on a 2% agarose gel.

### Generation of capped RNA

Capped RNA encoding dnSTAT3 was generated by the linearization of pcDNA3-STAT3-Y705F (Addgene no. 74434), which has been previously validated as dominant negative (10) using *Dra*III and subsequent *in vitro* transcription using the T7 mMessage mMachine Kit of Ambion (cat. no. AM1344; Thermo Fisher Scientific, Inc.) following the manufacturer's instructions.

### Cloning

For the generation of new *in situ* probes, TOPO TA cloning was performed (cat. no. K460001; Thermo Fisher Scientific, Inc.). As inserts, coding sequence fragments were generated using PCR using the Expand™ Long Template PCR-System (cat. no. 11681834001; Merck KGaA) as polymerase. Following an initial denaturation at 94°C for 1 min, 35 cycles of 12 sec at 94°C, 15 sec at 56°C and 30-60 sec elongation at 68°C were run followed by an additional 3 min at 68°C. Primers and GenBank accession numbers are listed in Table I. A 1400 fragment of *trpm7* from clone cb495 provided by ZIRC was subcloned via *Bam*HI and *Eco*RI into pCS2+. *In situ* probes were then generated by the *in vitro* transcription of linearized plasmids using SP6, T3 or T7 polymerase (NEB cat. nos. M0207, M0378 and M0251), respectively and the RNA DIG labeling mix (cat. no. 11277073910; Merck KGaA).

A plasmid encoding human GRK4 ΔNLS, in which amino acids 224-226 were mutated from KKR to LLE (Table II), was generated by site-directed mutagenesis from pCS2+Flag-hGRK4 (4).

### In situ hybridization

Embryos were fixed in 4% buffered paraformaldehyde (cat. no. P6148; Merck KGaA) at 4°C overnight, dehydrated in a graded methanol series and stored at −20°C for at least 1 h before processing by *in situ* hybridization. *In situ* hybridization was performed with DIG-labeled *in situ* probes essentially as described in great detail in the study by Thisse and Thisse (11). Plasmids not listed above were kindly provided by Professor Jeroen Bakkers (*tbx2b*) (12) and ZIRC (*slc20a1a/*clone cb1011).

### Cells, cell culture and transfection

293T cells were cultured in high-glucose DMEM containing 10% heat-inactivated fetal bovine serum and antibiotics (all from Gibco; Thermo Fisher Scientific, Inc.). Transfections with a plasmid encoding C-terminally GFP-tagged GRK4 variants were performed in six-well plates using Lipofectamine 3000 according to the manufacturer's recommendations (cat. no. L3000008; Thermo Fisher Scientific, Inc.). After 1 day, the cells were transferred to poly-D-lysine-coated coverslips. At 2 days following transfection, the cells were processed for immunofluorescence.

The cells were routinely and regularly examined for mycoplasma contamination using the LookOut Mycoplasma PCR Detection kit and according to the manufacturer's recommendations (cat. no. MP0035; Merck KGaA). Cells were authenticated and matched to the Cellosaurus databases.

### Immunofluorescence

For the visualization of cilia and apical cell borders of the proximal tubule, embryos at 48 h post-fertilization (hpf) were fixed in 4% buffered paraformaldehyde at 4°C overnight. Subsequently, the staining protocol provided by Jaffe *et al* (13) was followed. The primary antibodies used were mouse anti-acetylated tubulin (1:500, cat. no. T6793; Merck KGaA) and rabbit anti-PKCz (1:500, cat. no. sc-216; Santa Cruz Biotechnology, Inc.). Alexa-coupled secondary antibodies were used for detection (1:1,000, cat. nos. A10037 and A11008; Invitrogen, Thermo Fisher Scientific, Inc.). Following immunostaining, the tail of the embryos was cut off using forceps and embedded between two coverslips using Vectashield with DAPI (cat. no. VEC-H-1200; Biozol). The same staining protocol was used to detect p-Stat3 (1:100, clone no. PS3/1; MBL International Corporation) and total STAT3 (1:500, cat. no. 10253-2-AP, Proteintech Group, Inc.; or 1:500, cat. no. ab226942, Abcam).

Transiently transfected 293T cells expressing GFP-tagged GRK4 variants were fixed for 10 min using 4% buffered paraformaldehyde and mounted using Vectashield with DAPI.

### Imaging

Living embryos were anesthetized by immersion in 0.02% (m/v) buffered tricaine and imaged with a Leica M125 stereomicroscope and a Leica IC80 HD camera or a Flexacam C1 color camera. The same setup was used for imaging embryos processed by *in situ* hybridization. A Leica M205FA and a K8 sCMOS camera were used for epifluorescence analyses. Confocal z-stacks were acquired with a Leica SP5 or a Leica Stellaris 5 system, respectively and maximum projections were generated.

### Measurements

FIJI (14) was used to perform all measurements: The line tool was used to measure the length of distinct pronephric tubule parts in *in situ* hybridizations and for the measurement of the proximal tubule diameter in maximum projections of z-stacks. The freehand selection tool was used to measure the area of the glomerulus in *in situ* hybridizations. Cilia length was determined using the Simple Neurite Tracer within the neuroanatomy tool set. In detail, the beginning and the end of a given cilium was selected in 3D and the whole length was traced and measured.

To assess p-Stat3 levels, all embryos processed by antibody staining were imaged and images were compared side-by-side for staining being stronger than the average control-injected embryo in each experiment.

### Statistical analysis

Statistical analyses were performed with the aid of Prism 10 (Dotmatics). First, data were analyzed for normal distribution using a Shapiro-Wilk-test before the respective parametric or non-parametric test was applied. An α level of 0.05 was considered to indicate a statistically significant difference. The specific tests applied, as well as P-values and experimental repeats are described in the respective figure legends. In detail, the following tests were applied for data with normal distribution: A two-tailed t-test with Welch's correction, one-way ANOVA with Sidak's multiple comparison test and two-way ANOVA with Sidak's multiple comparison post-test. Data not following a normal distribution were analyzed using the following tests: A two-tailed Mann-Whitney test or the Kruskal-Wallis test with Dunn's multiple comparison post-test. All Fisher's exact tests were two-sided and the α levels were Bonferroni-corrected for the number of comparisons. Values of P<0.025 were considered to indicate statistically significant differences in the case of two comparisons.

## Results

### Loss of Grk4 disrupts normal pronephros patterning

**Figure 1:**
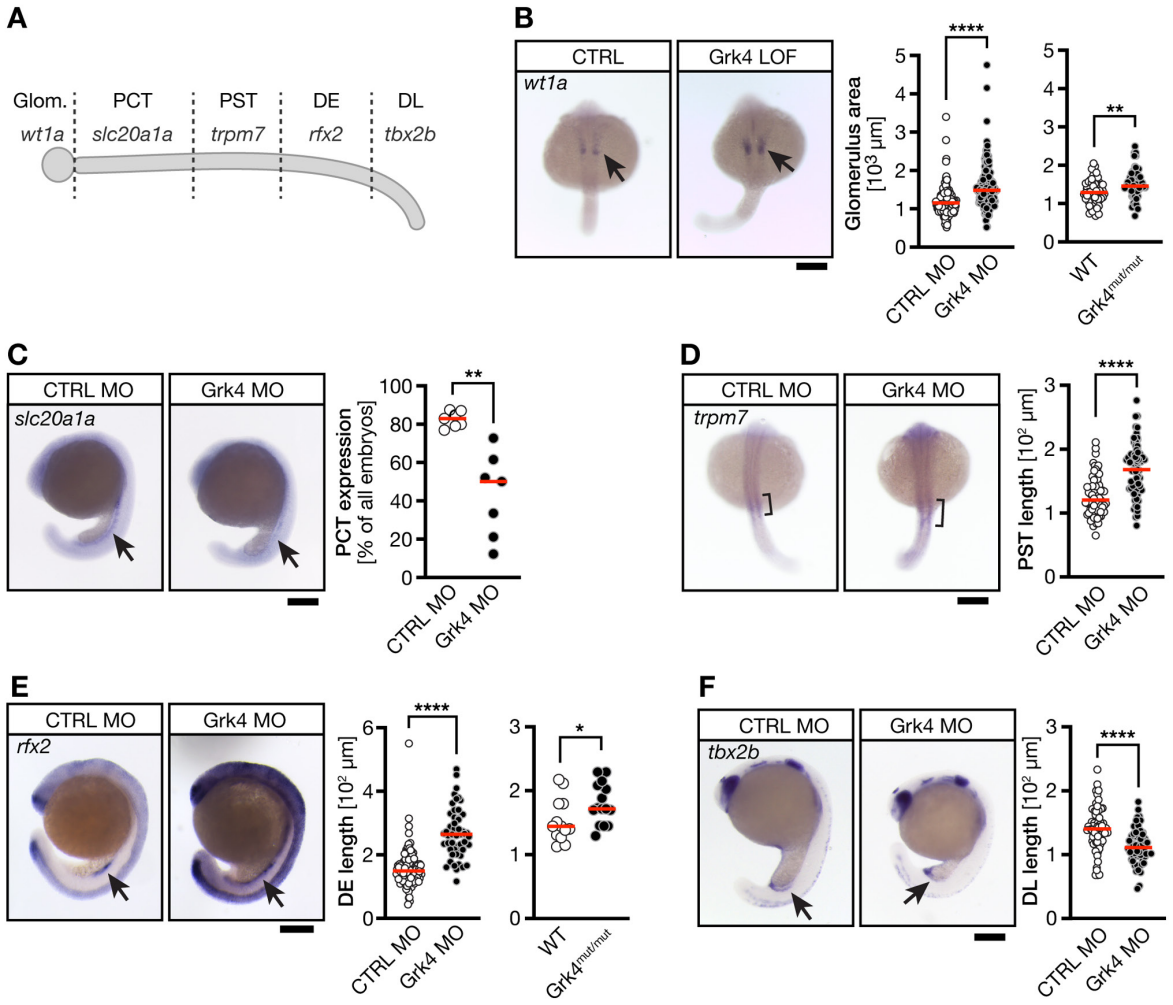
Loss of Grk4 disrupts normal pronephros patterning. (A) Cartoon illustrating the segments of the developing zebrafish pronephros and marker genes used to visualize the segments. (B) Loss of Grk4 increased the area of the glomeruli, as shown by *wt1a* expression. n=6 experiments with 150-192 embryos in total. ^****^P<0.0001 (data were analyzed using a two-tailed Welch's test). n=64 WT and 52 homozygous mutant embryos, respectively. ^**^P=0.0012 (data were analyzed using a two-tailed Welch's test). (C) Grk4 depletion limited the abundance of cells positive for the PCT marker *slc20a1a*. n=7 experiments with 118-143 embryos in total. ^**^P=0.0029 (data were analyzed using a two-tailed Welch's test). (D) Grk4 knockdown resulted in the elongation of the PST as shown by *trpm7* expression. n=3 experiments with 73-74 embryos in total. ^****^P<0.0001 (data were analyzed using a two-tailed Welch's test). (E) The DE segment, as detected by *rfx2* expression, was enlarged in Grk4 knockdown and mutant embryos compared to control embryos. n=3 experiments with 51-128 embryos injected with MOs in total. ^****^P<0.0001 (data were analyzed using a two-tailed Welch's test). n=13 WT and 15 homozygous mutant embryos, respectively. ^*^P=0.0451 (data were analyzed using a two-tailed Welch's test). (F) The DL segment was shortened upon Grk4 loss-of-function. n=3 experiments with 56-77 embryos in total. ^****^P<0.0001 (data were analyzed using a two-tailed Welch's test). All images: Whole mount *in situ* hybridization at 20-22 somite stage (ss). Arrows and brackets indicate the expression domain. Scale bars, 200 *μ*m. Glom, glomerulus; PCT, proximal convoluted tubule; PST, proximal straight tubule; DE, distal early tubule; DL, distal straight tubule; Grk4, G protein-coupled receptor kinase 4; CTRL, control; MO, morpholino oligonucleotide; WT, wild-type.

Zebrafish serve as a simple, yet elegant model of human kidney development, as they develop only two nephrons, one on each side of the midline. The zebrafish pronephros furthermore has a very similar composition as the mammalian nephron and displays a comparable functionality during embryonic development (15). In the present study, in order to assess Grk4-dependent kidney development in greater detail, two models were used, which were previously validated and analyzed by the authors: A knockdown approach by an antisense MO injection into the yolk of fertilized eggs and a mutant, in which two amino acids at position 194 and 195 are deleted producing a hypomorphic allele (4). Whole mount *in situ* hybridization for commonly used marker genes was performed to visualize distinct regions of the pronephros (Fig. 1A): Using a probe against *wt1a* revealed an enlargement of the glomerular area in Grk4-deficient embryos compared to control embryos (Fig. 1B), whereas the length of the proximal convoluted tubule, as detected by *slc20a1a* expression, was reduced (Fig. 1C). By contrast, the depletion of Grk4 increased the length of the proximal straight tubule, which corresponds to the proximal tubule in the mammalian nephron (Fig. 1D). A stronger signal of *rfx2* (Fig. 1E) was also observed at the expense of the distal late tubule visualized by *tbx2b* expression (Fig. 1F). Taken together, these results suggest that Grk4 is involved in pronephros patterning.

### Grk4 LOF results in the upregulation of Nhe3a

**Figure 2:**
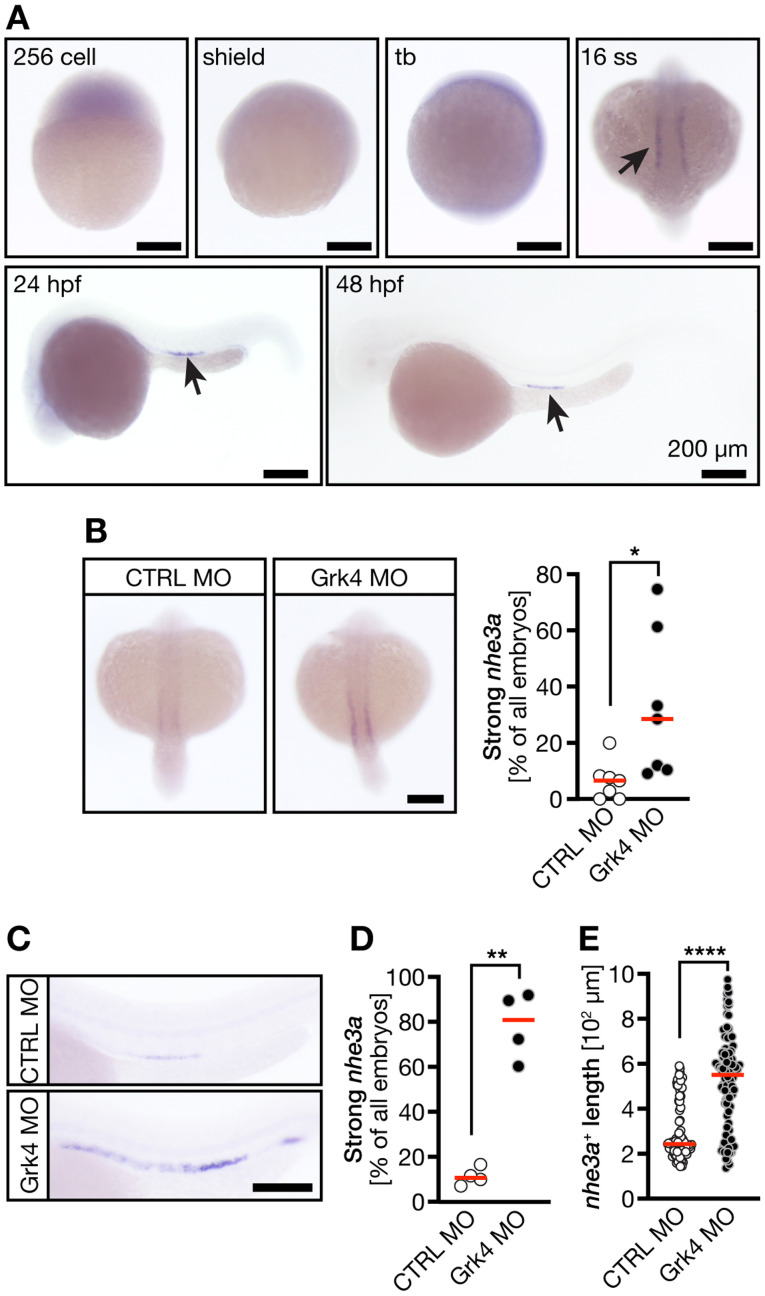
Grk4 loss-of-function results in the upregulation of *nhe3a*. (A) Detection of nhe3a expression during early zebrafish development using *in situ* hybridization. Expression (blue, indicated by arrows) can be seen in pronephric mesoderm at 16 ss and proximal tubule cells at 24 and 48 hpf. (B) At 20ss, *nhe3a* was more highly expressed in Grk4 morphant embryos than in the controls. Graph illustrates the percentage of embryos with strong *nhe3a* signals. n=7 experiments with 121-166 embryos in total. ^*^P=0.0374 (data were analyzed using a two-tailed Welch's test). (C) Representative images illustrating *nhe3a* expression in control and Grk4 MO-injected embryos at 48 hpf. (D) The percentage of embryos exhibiting a strong *nhe3a* expression was higher in Grk4 knockdown embryos. n=4 experiments with 91-103 embryos in total. ^**^P=0.0019 (data were analyzed using a two-tailed Welch's test). (E) *Nhe3a* signals can be detected over a longer region in Grk4-depleted embryos than in the controls at 48 hpf. n=4 experiments with 89-102 embryos in total. ^****^P<0.0001 (data were analyzed using a two-tailed Welch's test). Scale bars, 200 *μ*m. ss, somite stage; hpf, hours post-fertilization; Grk4, G protein-coupled receptor kinase 4; CTRL, control; MO, morpholino oligonucleotide.

In mammals, GRK4 modulates renal sodium reabsorption through dopamine receptor desensitization in the proximal tubule (16,17). As an extension of the proximal straight tubule was observed in Grk4-knockdown embryos, the authors wished to determine whether Nhe3a, the sodium transport protein, controlled by dopamine receptors in the mammalian proximal tubule (18), was affected by Grk4 LOF. Hence, *in situ* hybridization was performed at several developmental stages for *nhe3a*, the close zebrafish homolog of mammalian NHE3. It was observed that *nhe3a* is maternally provided and ubiquitously expressed, as shown for the 256 cell stage (Fig. 2A), but appears to be downregulated when embryonic gene expression begins; thus, at the shield stage, almost no expression remains. Towards the end of gastrulation (tailbud stage), and with the beginning of somitogenesis (as shown for the 16 somites stage), *nhe3a* begins being expressed again. From these stages on, when the pronephric mesoderm has formed, *nhe3a* can be exclusively detected in pronephric cells with the highest abundance in a region corresponding to the proximal straight tubule in wild-type embryos and also in the distal late segment or the cloaca, respectively (Fig. 2A). In embryos lacking functional Grk4, however, the expression domain of *nhe3*a was elevated at the 20 somites stage (Fig. 2B). At 48 hpf, when the pronephros has become functional, a strong *nhe3a* expression and a larger expression domain along the pronephric tubule could be detected (Fig. 2C). Hence, Grk4 appears to regulate Nhe3a expression during kidney development.

### Nhe3 inhibition rescues kidney patterning and ciliopathy phenotypes

**Figure 3:**
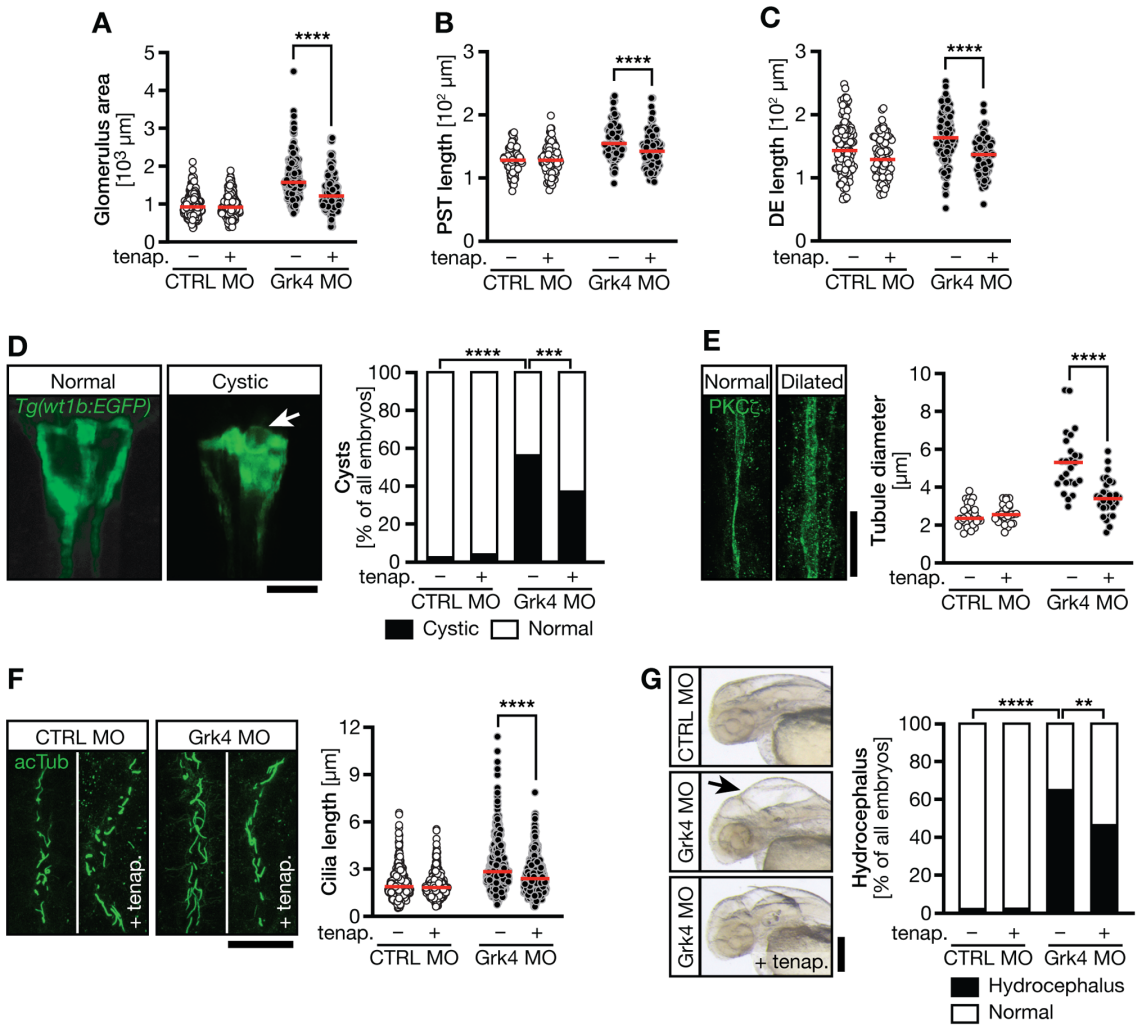
Nhe3 inhibition rescues kidney patterning and ciliopathy phenotypes. (A) Tenapanor treatment reduced the size of the *wt1a* expression domain in Grk4 knockdown embryos indicating a smaller glomerulus size. n=4 experiments with 218-242 embryos in total. ^****^P<0.0001 (data were analyzed using two-way ANOVA with Sidak's multiple comparison post-test). (B) The length of the PST, as detected by *trpm7* expression, was reduced in Grk4 morphants by tenapanor. n=3 experiments with 90-100 embryos in total. ^****^P<0.0001 (data were analyzed using two-way ANOVA with Sidak's multiple comparison post-test). (C) Tenapanor also decreased the DE length of Grk4 knockdown embryos. n=3 experiments with 74-123 embryos in total. ^****^P<0.0001 (data were analyzed using two-way ANOVA with Sidak's multiple comparison post-test). (D) Fluorescence image illustrating the glomerulus and parts of the proximal tubules in *Tg(wt1b:GFP)* transgenic embryos. Arrows indicate cystic glomeruli, which occur more often in Grk4 MO embryos than in control-injected embryos. Tenapanor reduced the percentage of embryos developing cysts. n=5 experiments with 144-166 embryos in total. ^***^P=0.0003 and ^****^P<0.0001 (data were analyzed using a two-sided Fisher's exact test with the Bonferroni correction applied). (E) Confocal z-stacks of pronephric tubules stained using anti-PKCz antibody. Grk4 knockdown produces dilated pronephric tubules. Tenapanor reduces the extent of dilatation. n=3 experiments with 25-29 embryos in total. ^****^P<0.0001 (data were analyzed using two-way ANOVA with Sidak's multiple comparison post-test). (F) Confocal stacks of cilia visualized by immunofluorescence using an acetylated tubulin (acTub) antibody. Tenapanor also reduced the length of cilia in the distal pronephric tubules in Grk4-depleted embryos. n=3 experiments with 446-639 cilia in total. ^****^P<0.0001 (data were analyzed using the Kruskal-Wallis test with Dunn's multiple comparison post-test). (G) Hydrocephalus (arrow) formation in Grk4-depleted embryos was significantly reduced upon treatment with tenapanor. n=5 experiments with 150-167 embryos in total. ^**^P=0.0011 and ^****^P<0.0001 (data were analyzed using a two-sided Fisher's exact test with the Bonferroni correction applied). Analyses were performed at (A-C) 20 ss and (D-G) 48 hpf. Scale bars: (D and G) 200 *μ*m, and (E and F) 20 *μ*m. Nhe3, sodium-hydrogen exchanger 3; Grk4, G protein-coupled receptor kinase 4; CTRL, control; MO, morpholino oligonucleotide; ss, somite stage; hpf, hours post-fertilization; tenap., tenapanor.

To examine whether the observed *nhe3a* upregulation contributes to the pronephros phenotypes evoked by Grk4 LOF, experiments using the specific NHE3 inhibitor, tenapanor, were performed. Zebrafish eggs were injected with either a control or the Grk4 MO and subsequently treated during organogenesis with 5 *μ*M tenapanor or the vehicle. Of note, tenapanor partially, yet significantly, rescued the disrupted kidney patterning, as shown for the expression domains of *wt1a*, *trpm7* and *rfx2* (Fig. 3A-C). Furthermore, significantly fewer Grk4-knockdown embryos developing cystic glomeruli were observed compared to vehicle-treated counterparts (Fig. 3D), with less dilated pronephric tubules (Fig. 3E) and the significant shortening of pronephric cilia (Fig. 3F). In addition, hydrocephalus, which develops as a consequence of the pronephric dysfunction (4), occurred to a lesser extent when Grk4 knockdown embryos were treated with tenapanor (Fig. 3G). By contrast, in the control-injected embryos, tenapanor had no or only very minor effects on the assessed phenotypes. Taken together, these data suggest a Grk4-Nhe3a axis contributing to the Grk4 LOF phenotype in zebrafish embryos.

### Nhe3 upregulation in the absence of Grk4 is not mediated by dopaminergic receptor signaling

**Figure 4:**
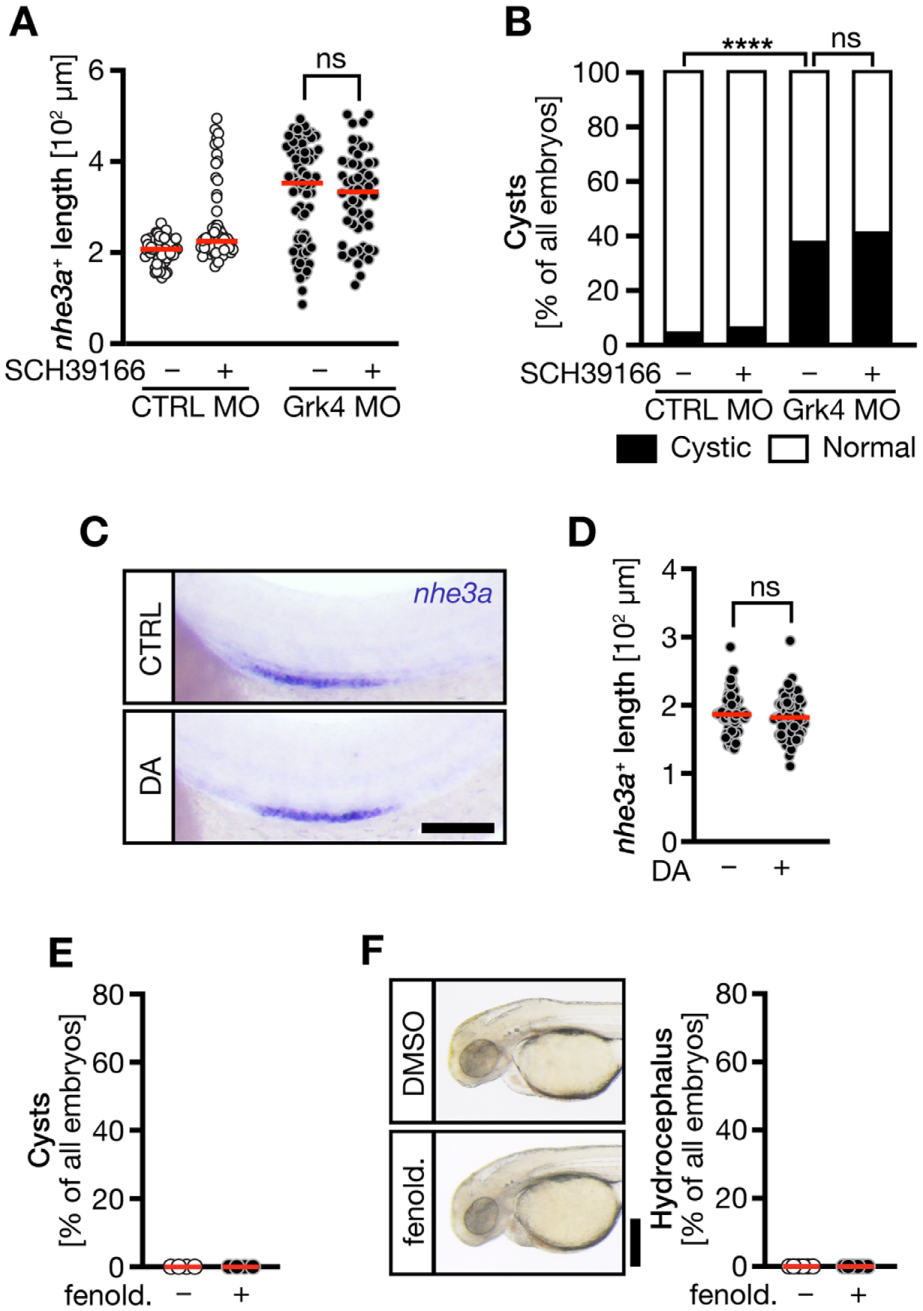
Modulation of dopaminergic receptor activity does not rescue Grk4 LOF kidney phenotypes. (A) Antagonism of dopaminergic receptor by SCH39166 failed to shorten the *nhe3a* expression domain. n=3 experiments with 54-67 embryos in total. ns; P>0.9999 data were analyzed using the Kruskal-Wallis test with Dunn's multiple comparison post-test). (B) The dopamine receptor antagonist, SCH39166, did not reduce the percentage of Grk4 LOF embryos developing glomerular cysts. n=4 experiments with 134-155 embryos in total. ^****^P<0.0001 ns, P=0.6176 (data were analyzed using a two-sided Fisher's exact test with the Bonferroni correction applied). (C) *Nhe3a* expression in embryos treated with the vehicle (CTRL) or dopamine. Scale bar, 200 *μ*m. (D) Dopamine administration did not alter the length of the *nhe3a* expression domain in Grk4-depleted embryos. n=3 experiments with 64-72 embryos in total. ns, P=0.3242 (data were analyzed using a two-tailed Welch's test). (E) Fenoldopam treatment did not induce cyst formation. n=4 experiments with 94-96 embryos in total. (F) Stimulation of dopaminergic receptor using fenoldopam did not result in hydrocephalus formation. n=6 experiments with 134-135 embryos in total. ns, not significant; DA, dopamine; Grk4, G protein-coupled receptor kinase 4; LOF, loss-of-function; Nhe3, sodium-hydrogen exchanger 3; Grk4, G protein-coupled receptor kinase 4; CTRL, control; MO, morpholino oligonucleotide; fenold., fenoldopam.

Subsequently, the present study assessed whether the observed phenotypes were coupled to dopaminergic receptor activity as Grk4 LOF may prevent receptor desensitization and cause elevated dopaminergic receptor signaling. Fertilized eggs were injected with control or Grk4 MO and treated with the vehicle or 10 *μ*M of the dopamine receptor antagonist, SCH39166, from the tailbud stage on until analysis at 48 hpf. Notably, no significant change was detected in the expression domain of *nhe3a* in either the control-injected or Grk4 MO-injected embryos (Fig. 4A). SCH39166 also failed to reduce the percentage of embryos developing glomerular cysts (Fig. 4B). To further exclude unleashed dopaminergic receptor signaling as a driver of aberrant pronephric development, wild-type zebrafish embryos were treated with dopamine. Yet again, the length of the *nhe3a*^+^ tubule was not altered (Fig. 4C and D). In addition, dopaminergic receptor stimulation using fenoldopam did not induce glomerular cysts or hydrocephalus (Fig. 4E and F). Hence, defects in pronephric development and function in Grk4-depleted embryos are unlikely due to abnormal dopaminergic receptor signaling.

### Nuclear localization of GRK4 is not required for normal pronephros development

**Figure 5:**
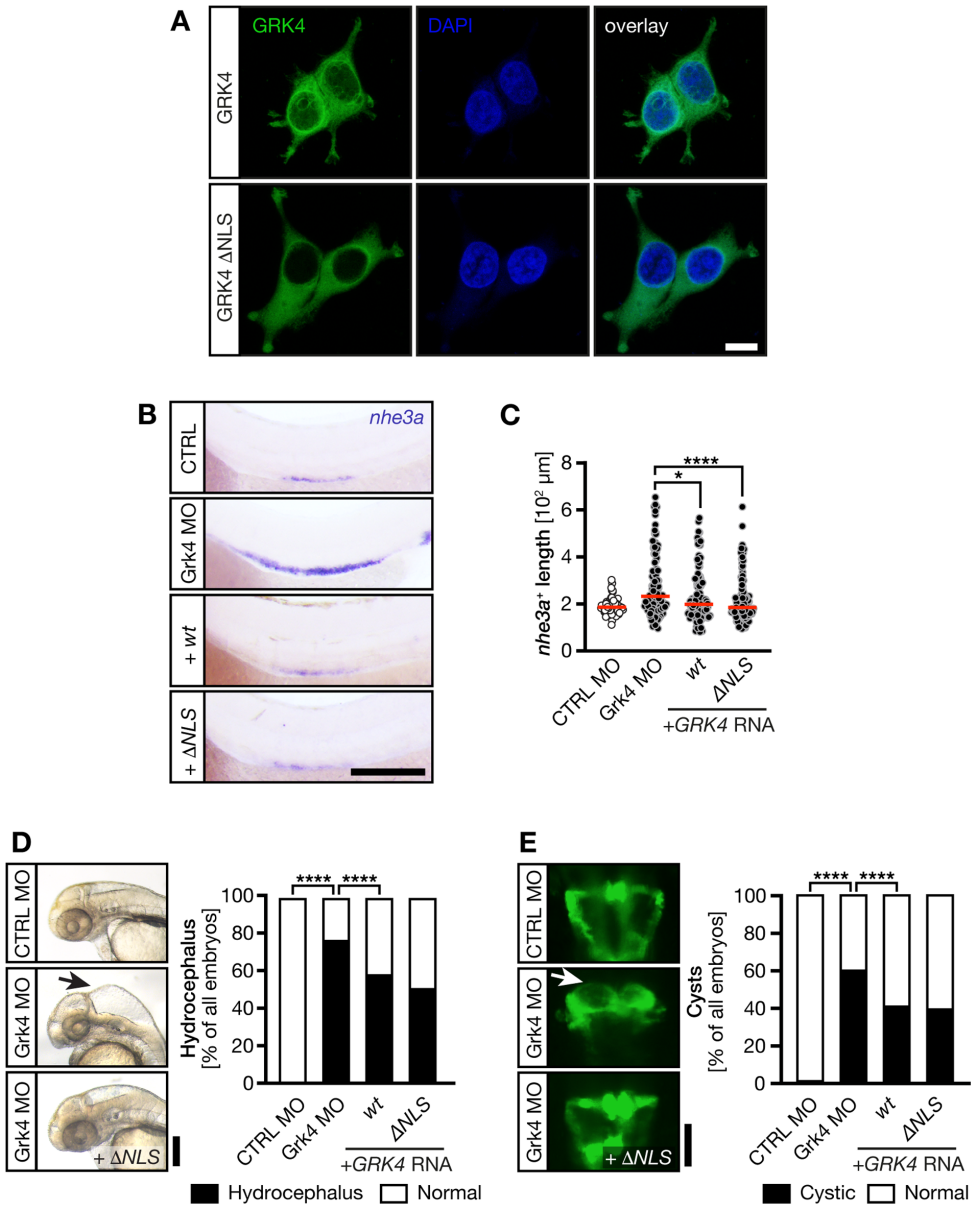
The actions of Grk4 during kidney development do not rely on its nuclear localization. (A) Confocal sections of 293T cells transiently transfected with GFP-tagged human GRK4 and GRK4 ΔNLS. Scale bar, 10 *μ*m. (B) Both wild-type GRK4 and GRK4 ΔNLS restored normal *nhe3a* expression in Grk4-depleted embryos. Scale bar, 200 *μ*m. (C) Analysis of the length of the *nhe3a*-positive pronephric tubule. n=4 experiments with 71-115 embryos in total. ^*^P=0.0147 and ^****^P<0.0001 (data were analyzed using the Kruskal-Wallis-test with Dunn's multiple comparison test). (D) Wild-type GRK4 and GRK4 ΔNLS rescue hydrocephalus formation in Grk4 MO-injected embryos. n=6 experiments with 161-175 embryos in total. ^****^P<0.0001 (data were analyzed using a two-sided Fisher's exact test with the Bonferroni correction applied). Scale bar, 200 *μ*m. (E) Glomerular cyst formation in Grk4 LOF embryos is reduced by wild-type GRK4 and GRK4 ΔNLS. n=8 experiments with 173-217 embryos in total. ^****^P<0.0001 (data were analyzed using a two-sided Fisher's exact test with the Bonferroni correction applied). Scale bar, 100 *μ*m. Grk4, G protein-coupled receptor kinase 4; NLS, nuclear localization sequence; CTRL, control; MO, morpholino oligonucleotide; wt, wild-type.

Previously and consistent with the data shown herein for dopaminergic receptor signaling, the authors found that the function of GRK4 during pronephros development does not depend on its kinase activity (4). In addition to the kinase domain, however, GRK4 also contains a nuclear localization sequence (NLS), which entails a low, yet reproducible GRK4 expression in the nucleus of transfected 293T cells (Fig. 5A). Mutation of the NLS (GRK4 ΔNLS) results in the exclusion of GRK4 from the nucleus and sole cytosolic distribution (Fig. 5A). Of note, rescue experiments with GRK4 ΔNLS normalized the expression of *nhe3a* and reduced both hydrocephalus, as well as cyst formation in Grk4 LOF embryos, suggesting an improvement in kidney function (Fig. 5B-E). Therefore, it can be concluded that the location of Grk4 action resides in the cytosol and does not depend on the nuclear localization of Grk4.

### Loss of Grk4 results in Stat3 activation

**Figure 6:**
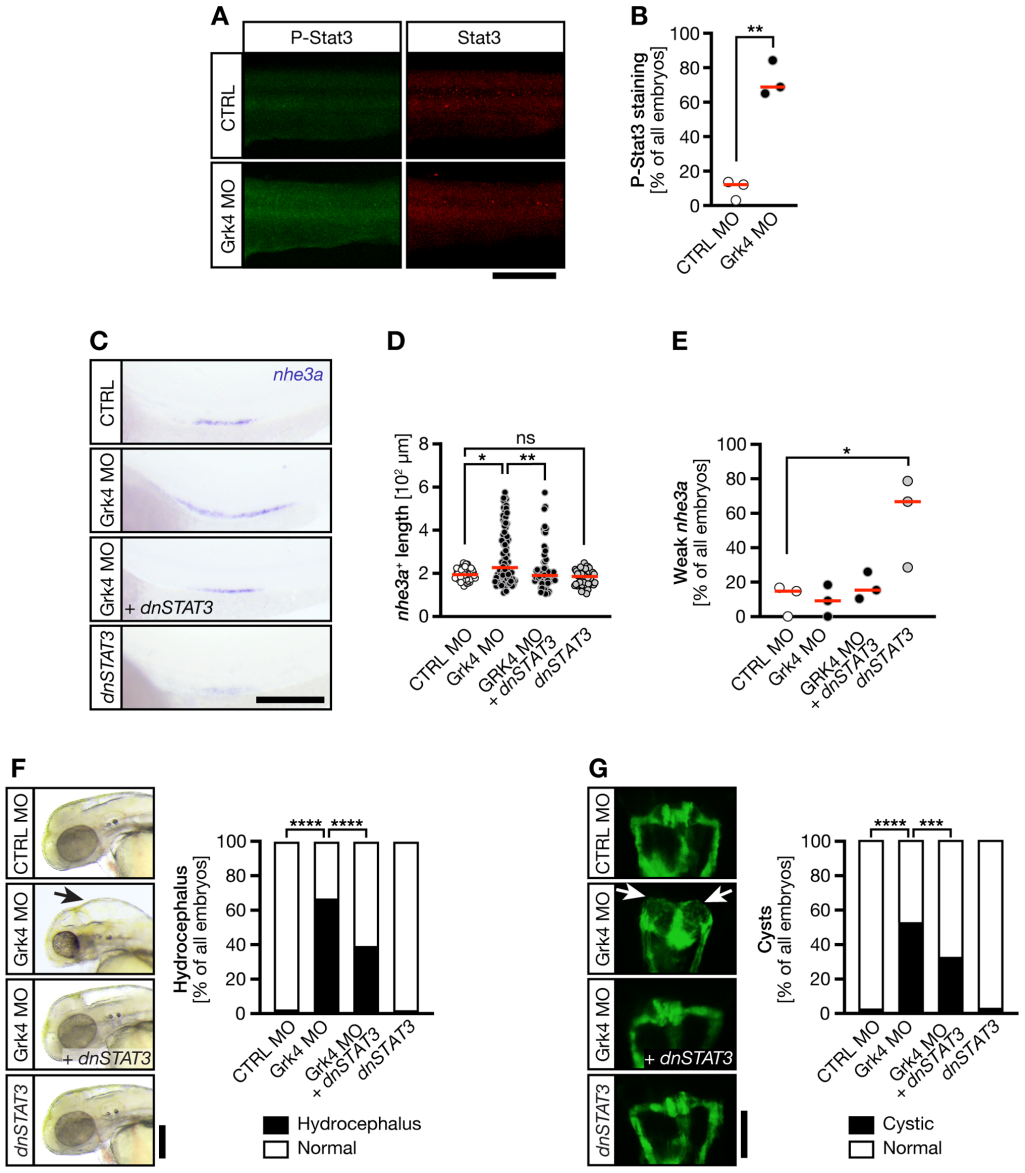
Pronephric Grk4 phenotypes are mediated by STAT3. (A) Representative immunofluorescence images of 24 hpf embryos stained for p-Stat3 and total Stat3. Scale bar, 200 *μ*m. (B) Loss of Grk4 increased the percentage of embryos exhibiting stronger p-Stat3 staining than the average control-injected embryo. n=3 experiments with 55-87 embryos in total. ^**^P=0.0022 (data were analyzed using a two-sided Welch's test). (C) *In situ* hybridization demonstrating *nhe3a* expression at 48 hpf. Scale bar, 200 *μ*m. (D) Expression of dnSTAT3 rescued the length of *nhe3a* expression in Grk4-depleted embryos. n=3 experiments with 59-89 embryos in total. ns, P=0.0588; ^*^P=0.0253 and ^**^P=0.0043 (data were analyzed using the Kruskal-Wallis-test with Dunn's multiple comparison test). (E) Embryos expressing dnSTAT3 displayed a weaker *nhe3a* signal. n=3 experiments with 59-89 embryos in total. ^*^P=0.0147 (data were analyzed usig one-way ANOVA with Sidak's multiple comparison test). (F) dnSTAT3 prevented hydrocephalus development upon Grk4 knockdown. n=4 experiments with 167-188 embryos in total. ^****^P<0.0001 (data were analyzed using a two-sided Fisher's exact test with the Bonferroni correction applied). Scale bar, 200 *μ*m. (G) Cyst formation was reduced upon dnSTAT3 expression. n=4 experiments with 167-188 embryos in total. ^***^P=0.0001 and ^****^P<0.0001 (data were analyzed using a two-sided Fisher's exact test with the Bonferroni correction applied). Scale bar, 100 *μ*m. ns, not significant; Grk4, G protein-coupled receptor kinase 4; STAT3, signal transducer and activator of transcription 3; dnSTAT3, dominant negative STAT3; CTRL, control; MO, morpholino oligonucleotide.

In addition to dopaminergic receptors, NHE3 has been reported to be controlled by other means. The transcriptional activity of STAT3 directs the RNA synthesis of *NHE3* in kidney epithelial cells by binding to its promoter (8). Moreover, STAT3 activity based on the phosphorylation of tyrosine 705 (corresponding to Y708 in zebrafish Stat3) is higher in cystic than in the healthy kidneys of patients diagnosed with autosomal polycystic kidney disease (19). Mutation of that same tyrosine 705 to phenylalanine abrogates the transcriptional activity of human STAT3 (20). Hence, the present study assessed STAT3 activity using an antibody specific for phosphorylated Stat3 in zebrafish and observed indeed a brighter fluorescence signal in Grk4-depleted embryos than in control embryos, while total Stat3 levels appeared to be the same (Fig. 6A and B). Co-injection of the Grk4 MO with RNA encoding for dominant negative STAT3 (dnSTAT3) revealed a significant reduction of the expression domain of *nhe3a* compared to embryos injected only with the Grk4 MO (Fig. 6C and D) and consistent with the transcriptional control of NHE3 by STAT3, many more embryos displaying a very weak *nhe3a* signal were counted (Fig. 6E). Last, but not least, the expression of dnSTAT3 partially, yet significantly reduced the percentage of embryos displaying kidney dysfunction as detected by hydrocephalus development (Fig. 6F) and glomerular cysts (Fig. 6G). Taken together, these findings suggest that the loss of Grk4 induces Stat3 activation in zebrafish embryos, which in turn results in higher rates of *nhe3a* transcription and subsequently the ciliopathy-like malformation of the developing pronephros, which was previously reported by the authors (4) and shown again herein.

## Discussion

The present study describes a novel function of GRK4 in facilitating normal nephron architecture. In the absence of functional Grk4, zebrafish embryos display alterations along the whole pronephros starting from the glomerulus, which becomes cystic thereafter. In addition, there is an enlargement of the segment, which corresponds to the proximal tubule in mammals. This is the region, where GRK4 desensitizes dopaminergic receptors in rodents and humans (21,22). The loss of Grk4 also increased the segment expressing *rfx2*, a gene, which is required for the development of multiciliated cells and the formation and extension of cilia (12,23). Hence, the elevated expression of *rfx2* may at least partially explain the expansion of pronephric cilia in number and length, which we have already previously reported in the absence of Grk4 (4). Furthermore, the present study observed the expression of the close homolog of mammalian NHE3 along the whole pronephric tubule instead of being concentrated in the proximal tubule. Similar to Rfx2, NHE3 proteins have been associated with cilia. In Xenopus epithelia, NHE3 proteins facilitate coordinated cilium formation and function of multiciliated cells (24). Interestingly, increased NHE3 levels have also been found in a mouse model of cilium dysfunction resulting in polycystic disease (25). Apart from its connection to cilia, NHE3 has been described as one of several sodium transport proteins regulated by dopaminergic receptors in the proximal tubule of mammals (16,18). Dopaminergic receptors inhibit NHE3 activity and prevent plasma membrane localization (26,27). Dopaminergic receptors further promote the proteasome-dependent degradation of NHE3 (28). The authors tried to investigate this in zebrafish. Notably though, both in the present study and in a previous study by the authors on Grk4, a dependency on dopaminergic receptors was not observed, which may also be a limitation of the model organism used. Consistent with the observation, however, a requirement of the kinase activity of Grk4 was also not found (4). As the authors unfortunately were unable to find an antibody reliably detecting zebrafish Nhe3a, the authors could not follow-up further on this and assess protein levels or subcellular localization, which would have added additional important insight into the regulatory impact of Grk4 on Nhe3. Since the upregulation of Nhe3a was detected on the level of mRNA, however, the authors considered a novel mechanism by which GRK4 regulates NHE3 proteins. GRK4 as with other GRKs of this subfamily translocates to the nucleus (29), which was also observed in 293T cells. While GRK4 does not appear to bind to DNA directly (29), it could influence RNA levels also by other means, such as modulating transcription factors. However, as GRK4 abrogated of its nuclear localization was as efficient in rescuing the Grk4 LOF phenotypes as wild-type GRK4, this hypothesis was dismissed and the authors searched for other mechanisms of NHE3 induction. STAT3, which is a gene required for cell survival (8), can be activated independent of receptor activation (8). It is highly active in the developing rat kidney (30) and displays an elevated activity in epithelial cells derived from polycystic kidneys (19,31). The transcriptional activity of STAT3 results specifically in the upregulation of NHE3 in epithelial cells on the RNA level by binding to the NHE3 promoter (8). Consistent with this, the present study observed an elevated Stat3 activity, as detected by phosphorylation at tyrosine 708 using immunofluorescence. The authors also tried to corroborate these findings further using western blotting, which however failed to produce quantifiable bands. The overexpression of a dominant negative version of STAT3 did not only normalize Nhe3a expression in the model used herein, but also rescued the cyst phenotype and general pronephros function. Most likely, GRK4 does not regulate STAT3 directly through phosphorylation, as previous experiments by the authors using kinase-dead GRK4 demonstrated that GRK4 does not rely on its kinase function in order to limit cilium elongation and to facilitate normal kidney phenotypes (4). One possible mechanism may be that other GRKs of the same subfamily become activated in the absence of GRK4 and compensate. Hence, mice deficient in GRK4, 5 or 6 are viable, while double homozygous embryos are at least partially lethal (4,6). GRK5 is in fact expressed in the zebrafish pronephros (6) and it inhibits the mineralocorticoid receptor by phosphorylation (32), which has been shown to cause STAT3 phosphorylation at serine 727 (33). While the canonical activation of STAT3 resulting in nuclear translocation and transcriptional activity relies on tyrosine phosphorylation at position 705 in mammals and 708 in zebrafish, which was tested herein, there is also a non-canonical mechanism via the dephosphorylation of serine 727. When STAT3 is dephosphorylated at this position, it can also enter the nucleus and act as transcription factor (34). A closer analysis of the STAT3 phosphorylation state in future studies would be of interest, as it could dissect which residue would be regulated by which subtype of GRK.

Another avenue of STAT3 regulation may be via mTOR, which is negatively regulated by GRK4 (4). In zebrafish oocytes, mTOR phosphorylates STAT3 to control centrosome position and microtubule stability, which are both essential components of cilia (35). Apart from mTOR, GRK4 appears to also interact with Fyn-related kinase (FRK), a member of the SRC-like kinase family (36). SRC-like kinases, which are non-receptor tyrosine kinases, are typical upstream activators of STAT3 (37). Gain-of-function variants of FRK have been associated with increased phosphorylation of STAT3 at the same tyrosine as we tested in here (38) and SRC kinase inhibitors, such as bosutinib, which also blocks FRK activity, can limit cyst growth in a murine model of polycystic kidney disease (39). Last but not least, cilia on their own have been reported to control STAT3 activity so that it is also possible that Grk4 loss indirectly leads to STAT3 activation through induction of cilium elongation, which is known to cause cilium dysfunction.

**Figure 7:**
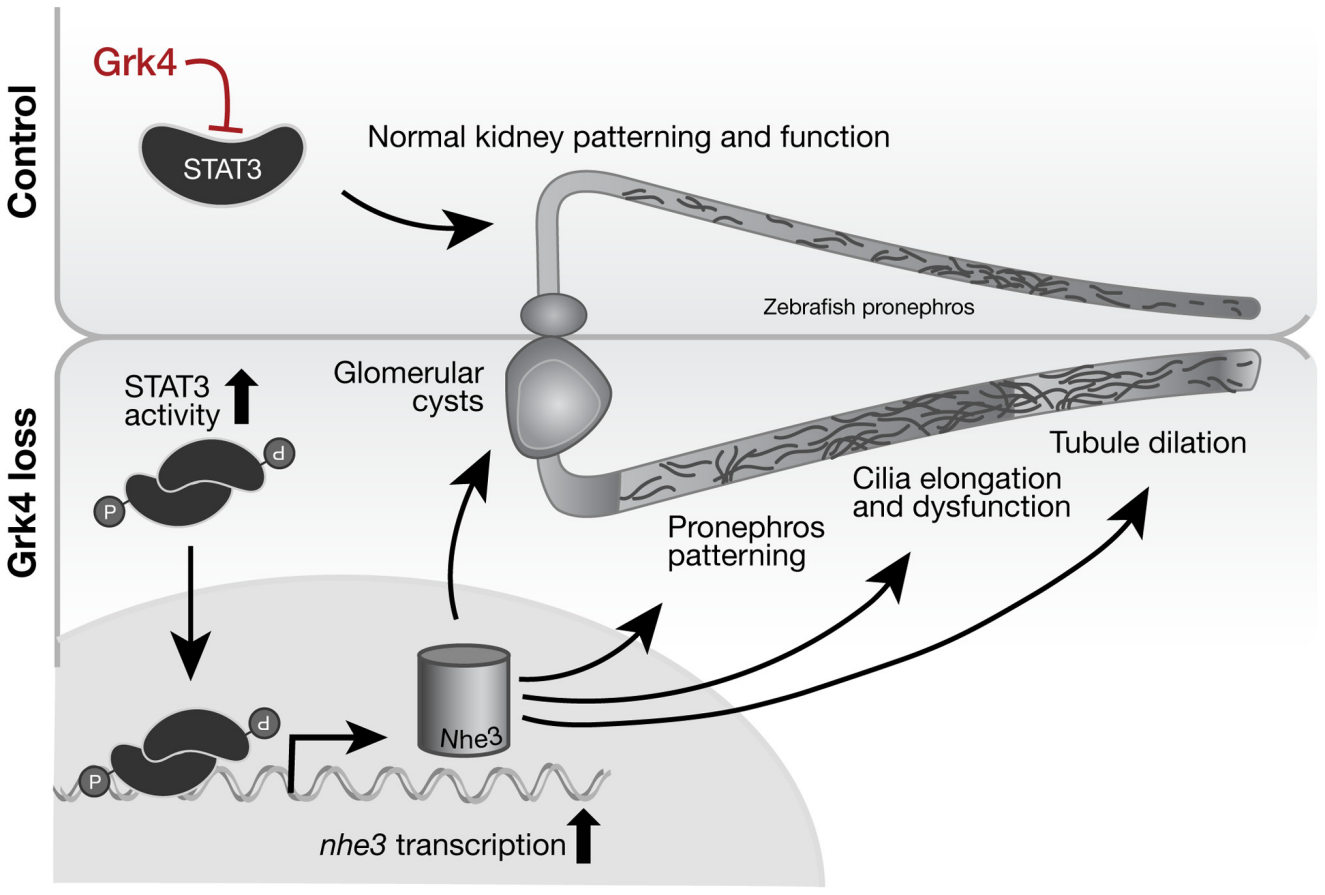
Schematic diagram providing a summary of the results of the present study. Proposed model of the newly identified Grk4-Stat3-Nhe3 pathway during zebrafish pronephros development. Grk4, G protein-coupled receptor kinase 4; STAT3, signal transducer and activator of transcription 3; Nhe3, sodium-hydrogen exchanger 3.

Taken together, the present study expands the previous characterization of kidney phenotypes upon Grk4 abrogation in zebrafish embryos and provides evidence that early kidney development is impaired in the absence of Grk4. In addition, the present study identified a novel regulatory mechanism of NHE3 expression by GRK4, which is independent of the well-known function of GRK4 in dopaminergic receptor desensitization (Fig. 7). To date, to the best of our knowledge, no such observations have been reported in Grk4 KO mice, which may be explained by known compensatory actions of other GRKs (4). Moreover, as cysts, which can be already observed in zebrafish in the embryo, appear in patients suffering from autosomal dominant polycystic kidney disease mostly at an advanced age, it may have escaped detection on the studies done so far on GRK4 KO mice.

### Translational impact for and beyond polycystic diseases

The previously identified regulatory action of GRK4 on NHE3 subcellular localization through the phosphorylation of dopaminergic receptors has ranked GRK4 as a potential therapeutic target in hypertension. The data presented herein classify GRK4 additionally as a candidate gene in polycystic kidney diseases, such as nephronophtisis or autosomal dominant polycystic kidney disease. It further suggests that the mechanistic impact of GRK4 on NHE3 is considerably beyond G protein-coupled receptors and places GRK4 in the center of a much more complex signaling network, which includes STAT3. STAT3 is involved in a rather large variety of pathologies ranging from immune disorders and inflammation over cardiovascular diseases and neurodegenerative conditions to several types of cancer (37). Hence, potential therapeutic strategies targeting GRK4 may require careful design in order to avoid interference with the full GRK4 signaling network.

The present study has certain limitations, which should be mentioned. The data presented herein solely rely on zebrafish embryos. While the authors demonstrated in a previous study that the abrogation of GRK4 in human fibroblasts, as well as kidney organoids derived from murine inner medullary collecting duct cells results in the same cilia defects as in zebrafish embryos (4), further studies using NHE3-expressiong mammalian cells or animal models are warranted to test the conservation of the newly identified Grk4-Stat3-Nhe3 axis across species.

## Figures and Tables

**Table I:** GenBank IDs, fragment sizes and primer sequences used for TOPO TA cloning.

Gene	GenBank ID	Size (bp)	Forward primer (5' to 3')	Reverse primer (5' to 3')
*nhe3a*	NM_001113473	900	GTGGACCAGAGGCTGGAA	GAGTCTGTGGAGCAGGTGC
*rfx2*	NM_001013278.1	1,392	GCTCTGTCTTCATGGGCCTT	GGCTTCTCTCGCTCATCCTC
*wt1a*	NM_131046	1,038	ACCCTCATGAAGACCCTCACT	GATTCCTCTGGTGCATGTTGT

Nhe3, sodium-hydrogen exchanger 3.

**Table II:** Primers (5' to 3') for the site-directed mutagenesis of NLS.

Forward	CAAAAAAAAAGAATATTACTGGAGAAAGGTGAAGCTATG
Reverse	CATAGCTTCACCTTTCTCCAGTAATATTCTTTTTTTTTG

NLS, nuclear localization sequence.

## Data Availability

The data generated in the present study may be requested from the corresponding author.

## Abbreviations

hpf: hours post-fertilization
GPCR: G protein-coupled receptor
GRK4: G protein-coupled receptor kinase 4
Nhe3: sodium-hydrogen exchanger 3
NLS: nuclear localization sequence
STAT3: signal transducer and activator of transcription 3
dnSTAT3: dominant negative signal transducer and activator of transcription 3
